# Anti-Fungal (*Aspergillus fumigatus*) Activity of *Pseudomonas aeruginosa* in Cystic Fibrosis Synthetic Sputum

**DOI:** 10.3390/pathogens13100875

**Published:** 2024-10-07

**Authors:** Gabriele Sass, Satya Kethineni, David A. Stevens

**Affiliations:** 1 California Institute for Medical Research, San Jose, CA 95128, USA; gcmsass@gmail.com (G.S.); kethinenisatya@gmail.com (S.K.); 2 Division of Infectious Diseases and Geographic Medicine, Department of Medicine, Stanford University School of Medicine, Stanford, CA 94305, USA

**Keywords:** *Aspergillus fumigatus*, *Pseudomonas aeruginosa*, pyoverdine, fungal biofilm, synthetic sputum, cystic fibrosis, microbial interaction

## Abstract

*Aspergillus fumigatus* (Af) and *Pseudomonas aeruginosa* (Pa) are pathogens inhabiting the lungs of persons with cystic fibrosis (CF), or immune-compromised patients, causing or aggravating disease. We previously investigated their microbial interaction as well as susceptibility to anti-fungal drugs using RPMI medium (contains undetectable iron concentrations), as is standard for susceptibility testing. Here we investigated microbial interaction in synthetic sputum medium (SSPM), a complex mixture designed to mimic the milieu in CF lungs. SSPM contains Fe^2+^. Pa laboratory strain PA14 or PA14 siderophore mutant planktonic culture filtrate, prepared in RPMI or SSPM, were compared for inhibition of Af biofilm formation. SSPM enhanced bacterial and fungal growth and the production of the Pa molecules pyoverdine, phenazines, and rhamnolipids. Af was more susceptible to these molecules in SSPM (with the exception of pyoverdine). SSPM interfered with fungal susceptibility to pyoverdine. Studies with the mutant helped to reveal that the reduced anti-fungal activity of pyoverdine in SSPM appears to be compensated by higher production of other anti-fungal molecules, e.g., rhamnolipids, phenazines, and PQS, and higher Af sensitivity to these molecules. In summary, SSPM better defines Pa–Af intermicrobial competition in the milieu of CF lungs.

## 1. Introduction

Microorganisms interact, affect each other, and are affected by the microenvironment in their hosts. An example for such a microenvironment are the lungs of persons with cystic fibrosis (CF), a hereditary disease caused by mutations in the cystic fibrosis transmembrane conductance regulator (CFTR), a chloride channel on epithelial cells, with the loss or under-function leading to mucoid plaques in several organs [1,2,3]. In consequence, the lungs of persons with CF have a reduced ability to clear, e.g., pathogens and are therefore more susceptible to chronic infection. Prominent bacterial and fungal pathogens in the lungs of persons with CF are *P. aeruginosa* and *A. fumigatus*, respectively, which impair lung function, especially in co-infections [4,5]. Both pathogens compete for resources in their microenvironment, most notably for iron, which is rare in mucoid plaques and abundant in micro-hemorrhages, with both being present in CF lungs [6]. CF lungs tend to have a higher iron content than non-CF or healthy lungs [7]. Low-iron conditions trigger the production of siderophores by both *P. aeruginosa* and *A. fumigatus* and lead to competition [8]. RPMI-1640 medium (“RPMI”), a medium that does not contain detectable amounts of iron, has been extensively used in vitro to study bacteria, fungi, and microbial combinations, likely because it is fully defined (allowing controlled modifications of constituent contents for metabolic studies), and moreover, mammalian cells will grow in it, facilitating concurrent studies of such cells with microbes. RPMI medium is also used to determine fungal drug susceptibility in clinical tests, using the CLSI method [9].

The *P. aeruginosa* siderophore pyoverdine has been shown to possess anti-fungal activity in vitro [8], investigated in RPMI medium. Pyoverdine is not produced in RPMI medium complemented with ferric iron [10]. In iron-enriched RPMI medium, anti-fungal activity is partially taken over by the phenazine pyocyanin [10]. The relevance of pyoverdine or pyocyanin in intermicrobial competition in vivo is not yet fully understood. Other anti-fungal molecules in *P. aeruginosa* filtrates encompass PQS [11,12,13], rhamnolipids [12], 1-hydroxyphenazine [14,15,16,17], phenazine-1-carboxamide [15,16,17], and phenazine-1-carboxylic acid [17].

In CF lungs, the growth milieu for pathogens is complex, containing multiple amino acids and factors that RPMI does not provide. To investigate relevant microbes in CF in a milieu closer to that encountered in lungs in vivo, synthetic sputum media were developed [18,19,20,21,22,23,24]. It has been shown that such media affect the physiology of microbes differently than would be seen in RPMI [22,23]. Using one such medium, SSPM, in which *P. aeruginosa* can grow, we showed that *A. fumigatus* could grow, and in a cohort of CF and non-CF *A. fumigatus* isolates, there were differences in susceptibility to voriconazole, a widely used azole anti-fungal drug, when planktonic testing was compared to RPMI medium [24].

Here, we compare the bacterial growth as well as composition and anti-fungal activity of *P. aeruginosa* filtrates, prepared in SSPM or RPMI on *A. fumigatus* growing and forming biofilm.

## 2. Materials and Methods

### 2.1. Materials

2,3-bis(2-methoxy-4-nitro-5-sulfophenyl)-2H-tetrazolium-5-carboxanilide inner salt (XTT), menadione, and RPMI 1640 medium were purchased from Sigma-Aldrich (St. Louis, MO, USA). Synthetic sputum medium was prepared as described previously [20]. This fully defined medium, based on CF sputum analyses, was developed to nutritionally mimic CF sputum. It has the same average concentrations of ions, free amino acids, glucose, and lactate as CF sputum samples. It utilizes the same 3-(N-morpholino) propanesulfonic acid (MOPS) buffer as in RPMI 1640 medium to mimic the native buffering system of the lung. The iron content in RPMI 1640 medium was below the detection limit (<1 µM, measured by inductively coupled plasma optical emission spectroscopy by Paolo Visca, Rome, Italy, personal communication), whereas SSPM contained 3.6 µM of ferrous iron. Pure pyoverdine, pure pyocyanin, and pure PQS were purchased from Sigma-Aldrich (St. Louis, MO, USA). For all pure molecules, large batches of aliquots were prepared and frozen, and a fresh aliquot was used in each experiment.

### 2.2. Strains and Isolates

The use of all microbes in our laboratory was approved by the CIMR Biological Use Committee (approval no. 001-03Yr.19). Assays were performed using the microbes shown in Table 1.

### 2.3. Experimental Setup

**Figure 1:**
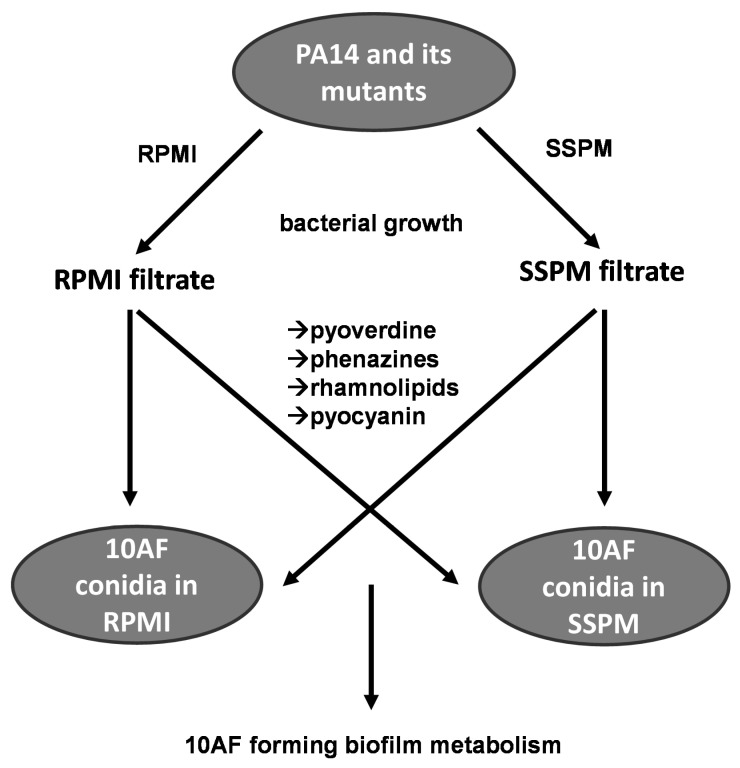
Experimental workflow diagram. Bacteria were grown in RPMI or SSPM at 37 °C and 100 rpm for 24 h before measuring growth. Sterile filtrates were prepared and tested for (arrows) pyoverdine, phenazine, rhamnolipid, and pyocyanin content as described in the Materials and Methods section. Effects of filtrates on fungal biofilm formation were tested in RPMI, SSPM, or a mixture of both media.

An overview of the experimental setup of this study is provided in Figure 1. Individual steps are described below.

### 2.4. Pseudomonas Filtrate Production

*P. aeruginosa* cultures were prepared by incubating 5 × 10^7^ cells/mL in RPMI 1640 medium (Sigma-Aldrich) or SSPM at 37 °C and 100 rpm for 24 h. Bacterial growth was measured at 610 nm using a spectrophotometer (Genesys 20, Thermo Fisher Scientific Inc., Waltham, MA, USA). Bacterial cultures were centrifuged at 200× *g* for 30 min at room temperature and filtered for sterility (0.22 μm).

### 2.5. Pyoverdine and Phenazine Measurement

Pyoverdine content in bacterial filtrates was measured at 405 nm as described previously using a spectrophotometer (Genesys 20) [31]. Pyoverdine was visualized under UV light and appeared as bright blue fluorescence. Phenazines were measured in bacterial supernatants at 360 nm using a spectrophotometer (Genesys 20). Pyoverdine and phenazine measurements were also normalized to bacterial growth using the following formula: Relative expression = OD405 or OD360/OD610. Biochemical measurement of the phenazine pyocyanin was performed as described previously [32] by chloroform extraction from bacterial filtrates and measurement in 0.2N HCl at 520 nm.

### 2.6. Biosurfactant Quantification on Oiled Paper

One sheet of standard printer paper was placed on a fat-free glass plate as described previously [33]. Briefly, the paper sheet was evenly covered with fluid mineral oil (sterile mineral baby oil; CVS, Woonsocket, RI, USA). Excess oil and air bubbles underneath the oiled paper were brushed off using lint-free tissue paper (Kimtech, Kimberley-Clark Worldwide, Inc, Roswell, GA, USA). Fifty-microliter droplets of bacterial filtrates were placed on the oiled paper in quadruplicate and allowed to spread and dry at room temperature. As controls, 50 µL droplets of the respective sterile media were placed on the same paper sheet. The diameters of the dried droplets were measured vertically and horizontally, as the drop shapes tended to be slightly irregular at higher biosurfactant concentrations. The diameters were used to calculate droplet areas, using the following formula: a (area) = r(radius)^2^π. Mean areas of sterile diluent were subtracted from areas produced by filtrates. Areas are displayed in mm^2^.

### 2.7. Visualization of Fungal Growth

10AF conidia were examined for differences in appearance in RPMI vs. SSPM after 2, 6, and 8 h of growth at 37 °C at 400× magnification (pictures taken by digital microscopy using an M83EZ-C02 microscope, OMAX Microscopes, Ningbo, China, in combination with OMAX Toup View Software, version 3.7). An example is shown in the Results section.

### 2.8. A. fumigatus Biofilm Formation Assay

Conidia (2.5 × 10^3^) were combined with *P. aeruginosa* filtrate or pure substance dilutions in a volume of 100 µL/well in a 96-well plate in RPMI medium or SSPM at the concentrations indicated in the figures and figure legends. RPMI 1640 medium or SSPM alone on the fungus served as the 100% metabolism control. Plates were incubated overnight at 37 °C before determining fungal metabolism as described below.

### 2.9. Assay for Measurement of Aspergillus Biofilm Metabolism

Biofilm formation was verified by optical microscopy before performing XTT assays. All experiments were evaluated by XTT metabolic assay as detailed previously [34,35]. Briefly, after the removal of all liquid from the plates, 150 µL of an XTT/menadione mixture (150 µg/mL XTT, 30 µM menadione) was added to each test well, and plates were incubated at 37 °C for about one hour. The liquid content from each well was transferred to a fresh well on a 96-well plate (100 µL) and assayed using a plate reader (Vmax, Molecular Devices, San Jose, CA, USA) at 490 nm. For better comparison, the metabolism of control wells (incubated with medium only, therefore having maximal possible fungal growth and metabolism) was considered to be 100%, and treated samples in the same medium were normalized to that.

### 2.10. Determination of the Isolate Dilution with 50% Anti-Fungal Activity (IC50)

Filtrates and pure molecules were diluted in their respective medium in 1:2 steps, with final concentrations indicated in the figures and figure legends. The concentration closest to inhibiting 50% of fungal metabolism was referred to as the IC50.

### 2.11. Statistical Analysis

Results were analyzed using Student’s *t* test if two groups were compared and 1-way ANOVA combined with Tukey’s post-test for multiple comparisons. All data in this study are expressed as mean ± SD. Data are also reported as the percent of the control. Each assay was performed with three to four biological replicates and three to four technical replicates. Representative experiments are shown.

## 3. Results

### 3.1. Comparison of Bacterial Growth and Pyoverdine Production by PA14 in RPMI Medium Compared to SSPM

**Figure 2:**
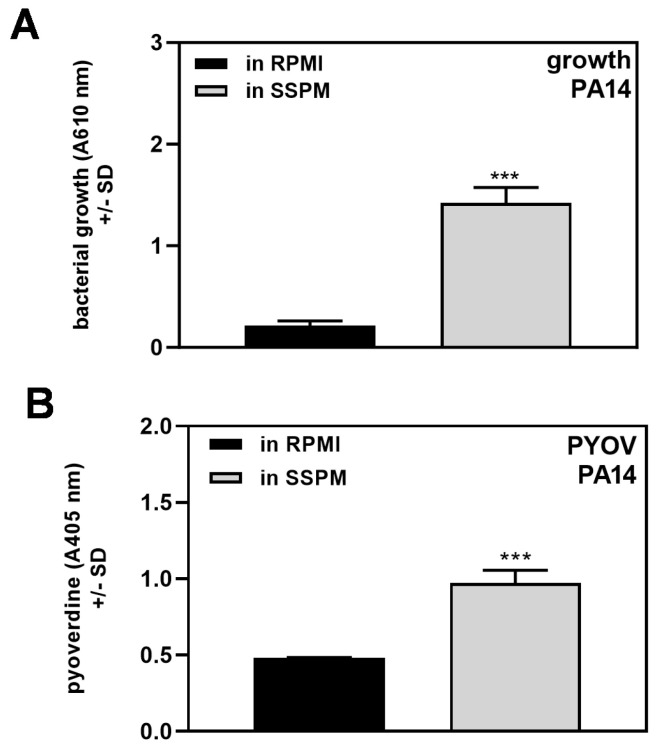
More growth and pyoverdine production by PA14 wild-type in SSPM. PA14 wild-type bacteria [5 × 10^7^ cells/mL] were incubated in RPMI or SSPM at 37 °C for 24 h. Bacterial growth was measured by absorption of the cultures at 610 nm vs. their respective sterile medium as a background control (**A**). Pyoverdine content in filtrates of these cultures was determined at 405 nm (**B**). Comparisons: RPMI (black bars) vs. SSPM (grey bars). Statistical analysis: unpaired *t*-test; three asterisks = *p* ≤ 0.001.

PA14 wild-type growth was initiated with equal numbers of bacteria in RPMI or SSPM. After 24 h of incubation, the growth in SSPM was about sevenfold higher than in RPMI (Figure 2A). The pyoverdine content also was higher in the SSPM filtrate compared to the RPMI filtrate (Figure 2B).

Normalization to bacterial growth in the respective media showed that individual bacteria produced less pyoverdine growing in SSPM than they did growing in RPMI (Table 2).

### 3.2. Earlier Fungal Hyphae Development and More Biofilm Metabolism in SSPM

**Figure 3:**
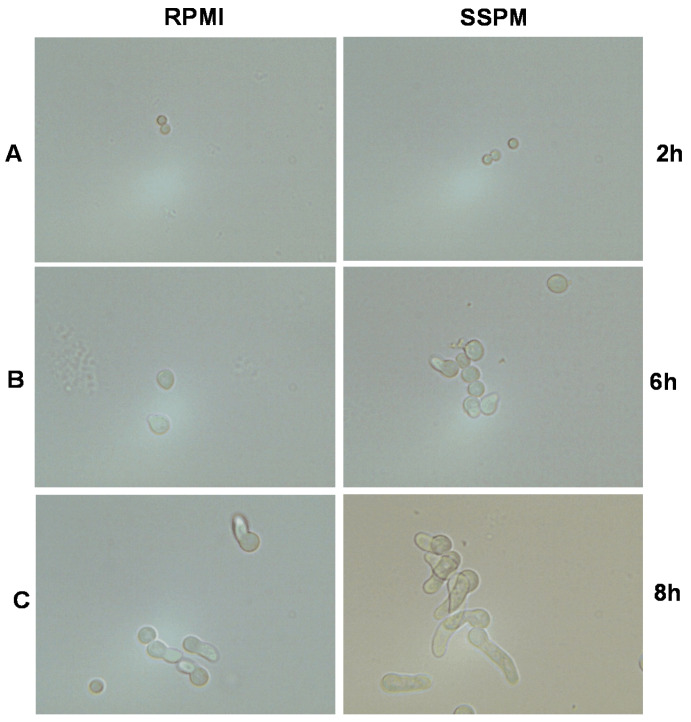
Earlier hyphae development in SSPM: 10AF structures growing from conidia were examined for differences in appearance after 2 (**A**), 6 (**B**), and 8 h (**C**) of growth at 37 °C at 400× magnification. For each timepoint and medium, a representative example picture is shown.

When conidia of *A. fumigatus* were cultured in RPMI or SSPM, we found no differences in morphology after two hours of incubation (Figure 3A). After 4 h of incubation, conidia in both cultures were enlarged, and conidia in SSPM cultures showed hyphal budding (Figure 3B). After 8 h of incubation, hyphae had started to form, with the RPMI culture still in early stages and the SSPM culture already showing hyphal growth (Figure 3C).

**Figure 4:**
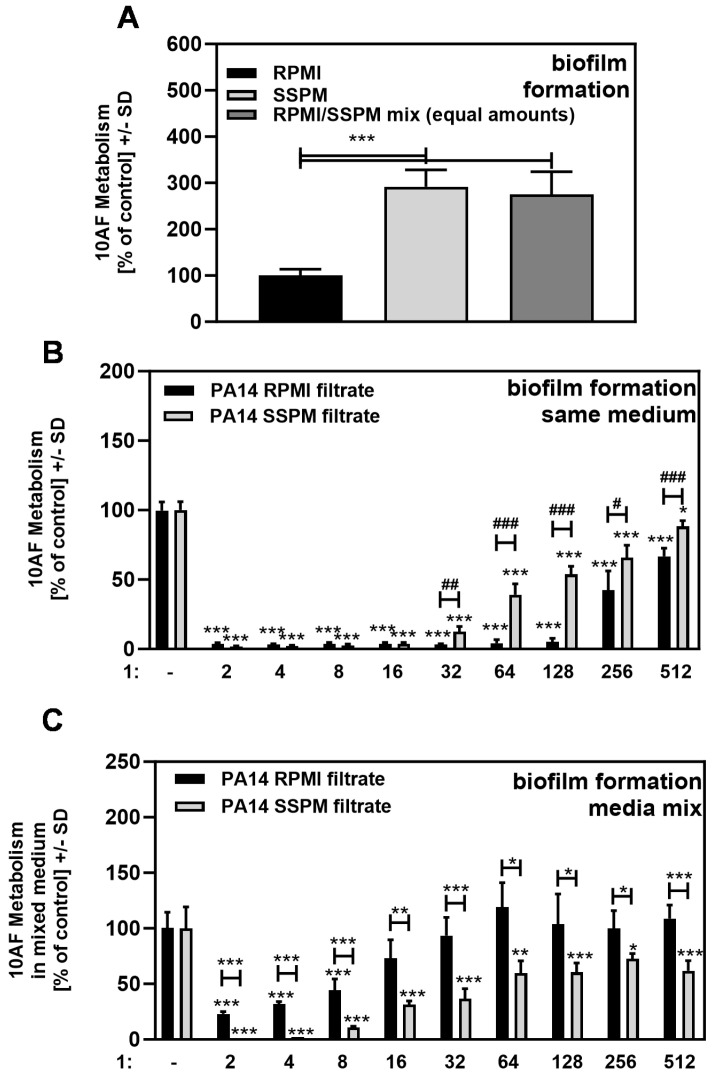
SSPM increases fungal biofilm metabolism and affects the anti-fungal activity of PA14 wild-type filtrates. 10AF conidia (10^5^ conidia/mL) were incubated without (**A**) or with (**B**,**C**) the addition of PA14 wild-type filtrate. Bacterial filtrates were diluted in 2-fold steps (final concentrations: 1:2 to 1:512). In (**B**), bacterial filtrates prepared in RPMI were combined with conidia suspensions in RPMI, and bacterial filtrates prepared in SSPM were combined with conidia suspensions in SSPM. In (**C**), bacterial filtrates prepared in RPMI were combined with conidia prepared in SSPM, and bacterial filtrates prepared in SSPM were combined with conidia prepared in RPMI. The volumes of filtrates and conidia suspensions were equal when combined. Assay plates were incubated at 37 °C for 16 h. Fungal metabolism was measured by XTT assay. Comparisons without brackets were performed by 1-way ANOVA. Metabolism in the presence of medium alone was regarded as 100% and compared to each filtrate dilution in the same medium. All other comparisons were performed by unpaired *t*-test as indicated by the ends of the brackets. In (**B,C**): filtrate in RPMI (black bar) vs. filtrate in SSPM (grey bars). Statistical analysis: one, two, or three asterisks or pound signs = *p* ≤ 0.05, or *p* ≤ 0.01, or *p* ≤ 0.001, respectively. Asterisks indicate decreases in fungal metabolism (= higher anti-fungal activity) and pound signs indicate increases in fungal metabolism (= less anti-fungal activity) in the bracketed pairs.

Consistent with the observations on growth in Figure 2, 10AF biofilm metabolism after 16 h of growth was significantly stronger in SSPM compared to RPMI (Figure 4A). We observed a 2–3 times higher metabolism in SSPM, and even in equal mixtures of SSPM and RPMI (Figure 4A).

### 3.3. Effects of PA14 Filtrate, Prepared in RPMI Medium or SSPM, on A. fumigatus 10AF Biofilm Formation

PA14 filtrate dilutions impaired fungal metabolism about equally up to concentrations of 1:16 when the assay was performed in the same medium in which the filtrate was prepared (RPMI or SSPM; Figure 4B). At higher dilutions, the anti-fungal activity of filtrates prepared in RPMI was significantly stronger than that of SSPM filtrates (Figure 4B), although the RPMI filtrates contained less of the strongest known pseudomonal anti-fungal molecule, pyoverdine (Figure 2B). This observation indicated that SSPM medium and pyoverdine might interact differently in some way from the interaction in RPMI, either by SSPM diminishing pyoverdine anti-fungal activity or by protecting the fungus from pyoverdine.

In order to diminish possible explanations, we first equalized the medium for the biofilm assay by combining the Pseudomonas filtrate that had been prepared in RPMI with conidia prepared in SSPM and vice versa, so that equal mixtures of RPMI and SSPM were present during each assay. Under these conditions, we observed that the mixture with the filtrate containing less pyoverdine (RPMI filtrate) was less anti-fungal than the mixture with the filtrate containing more pyoverdine (SSPM filtrate) (Figure 4C). However, under these conditions, both filtrates were less anti-fungal than they were in 100% of the medium in which they were prepared (compare Figure 4C to Figure 4B). In summary, pyoverdine might play an important role in the anti-fungal activity of *P. aeruginosa* not only when studied in RPMI but also when studied in a milieu mimicking natural conditions (SSPM), but SSPM might decrease the anti-fungal activity of pyoverdine.

### 3.4. Pyoverdine Anti-Fungal Activity Is Reduced by Ferrous Iron in SSPM

**Figure 5:**
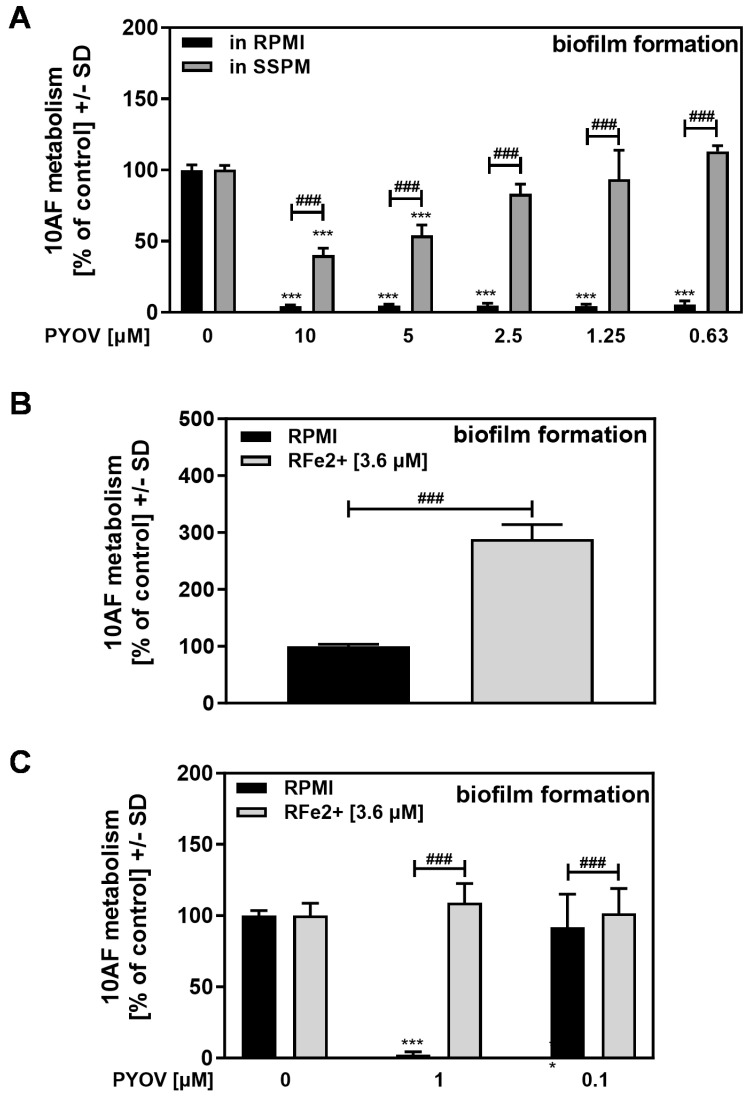
Pyoverdine anti-fungal activity is reduced by ferrous iron in SSPM. (**A**) 10AF conidia (10^5^ conidia/mL) were incubated in RPMI or SSPM with or without pyoverdine (PYOV; final concentrations 10–0.63 µM). (**B**,**C**) 10AF conidia (10^5^ conidia/mL) were incubated in RPMI or RPMI supplemented with ferrous iron (Fe^2+^; 3.6 uM) without (**B**) or with pyoverdine (**C**) (PYOV; final concentrations 1 or 0.1 µM). Assay plates were incubated at 37 °C for 16 h. Fungal metabolism was measured by XTT assay. Comparisons without brackets were performed by 1-way ANOVA. Metabolism in the presence of medium alone was regarded as 100% and compared in (**A**,**C**) to each pyoverdine dilution in the same medium. All other comparisons were performed by unpaired *t*-test as indicated by the ends of the brackets. Statistical analysis: three asterisks or pound signs = *p* ≤ 0.001. Asterisks indicate decreases in fungal metabolism (= higher anti-fungal activity) and pound signs indicate increases in fungal metabolism (= less anti-fungal activity) in the bracketed pairs.

Bacterial filtrates are a complex mixture of anti-fungal and potentially pro-fungal molecules, making it hard to ascribe net effects on fungal metabolism to just one molecule. To specifically compare the effects of pyoverdine on fungal biofilm formation between RPMI and SSPM, we used pure pyoverdine. Figure 5A shows that pure pyoverdine in RPMI has strong anti-fungal effects, at least down to 0.63 µM, whereas in the same assay, the anti-fungal activity of pyoverdine prepared in SSPM was much lower. One obvious difference between RPMI and SSPM is the presence of 3.6 µM ferrous iron in SSPM, whereas RPMI is virtually iron-free.

To decide if the presence of ferrous iron interfered with the anti-fungal activity of pyoverdine, we supplemented RPMI with 3.6 µM ferrous iron. Supplementation with ferrous iron increased the fungal biofilm metabolism in RPMI by about the same amount as seen for SSPM, compared to unmanipulated RPMI (compare Figure 5B to Figure 4A). Normalized to the unmanipulated fungal metabolism in the respective medium (= 100%), pyoverdine in RPMI ≥1 µM abolished biofilm metabolism, whereas ferrous iron in RPMI neutralized the anti-fungal activity of pyoverdine (Figure 5C).

**Figure A1:**
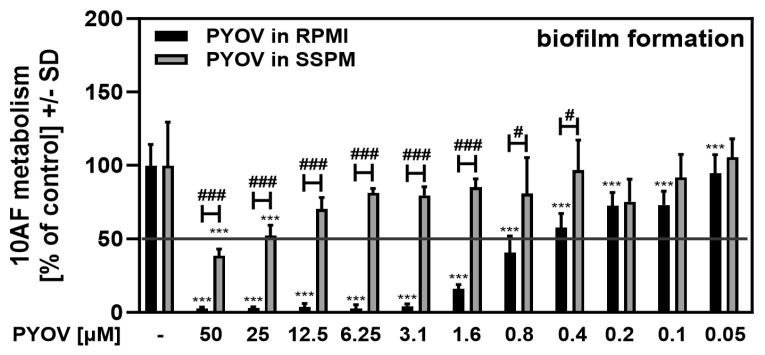
Anti-fungal activity of pure pyoverdine in RPMI and SSPM. 10AF conidia (10^5^ conidia/mL) were incubated with pure pyoverdine in RPMI or SSPM in 2-fold dilution steps from 50 to 0.05 µM. Assay plates were incubated at 37 °C for 16 h. Fungal metabolism was measured by XTT assay. Comparisons without brackets were performed by 1-way ANOVA. Metabolism in the presence of medium alone was regarded as 100% and compared to each pyoverdine dilution in the same medium. All other comparisons were performed by unpaired *t*-test as indicated by the ends of the brackets. Statistical analysis: one or three asterisks or pound signs = *p* ≤ 0.05 or *p* ≤ 0.001, respectively. Asterisks indicate decreases in fungal metabolism (= higher anti-fungal activity) and pound signs indicate increases in fungal metabolism (= less anti-fungal activity).

In conclusion, the ferrous iron in SSPM does not prevent the production of pyoverdine (Figure 2B) by *P. aeruginosa* but attenuates its anti-fungal effect (Figure 4B and Figure 5). This idea is supported by the finding that the IC50 for pyoverdine against 10AF conidia in RPMI was 0.4 to 0.8 µM, whereas the IC50 for pyoverdine in SSPM was 25 to 50 µM (Figure A1). Therefore, SSPM either protects the fungus or interferes with pyoverdine activity. Based on these results, it seems likely that the anti-fungal effects of the PA14 filtrate prepared and analyzed in SSPM (Figure 4B,C) must be partially based on the activity of molecules other than pyoverdine.

### 3.5. SSPM Induces the Production of Anti-Fungal Molecules Other than Pyoverdine

**Figure 6:**
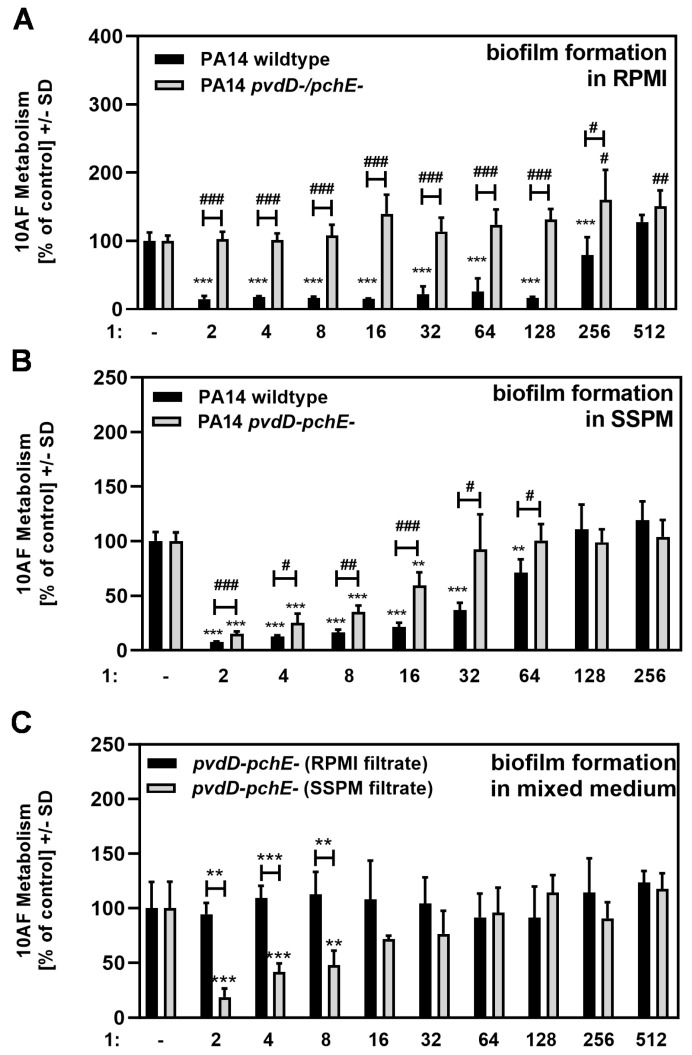
SSPM induces the production of anti-fungal molecules other than pyoverdine. 10AF conidia (10^5^ conidia/mL) were incubated with PA14 wild-type or a double siderophore mutant (PA14 pvdD-pchE-) filtrate in RPMI (**A**) or SSPM (**B**). PA14 pvdD-pchE- filtrate, prepared in RPMI or SSPM, was incubated in an equal mixture of RPMI and SSPM in 2-fold steps (final concentrations: 1:2 to 1:256 or 1:512) (**C**). Assay plates were incubated at 37 °C for 16 h. Fungal metabolism was measured by XTT assay. Comparisons without brackets were performed by 1-way ANOVA. Metabolism in the presence of medium alone was regarded as 100% and compared to each strain and filtrate dilution in the same medium. All other comparisons were performed by unpaired *t*-test as indicated by the ends of the brackets. Statistical analysis: one, two, or three asterisks or pound signs = *p* ≤ 0.05, *p* ≤ 0.01, or *p* ≤ 0.001, respectively. Asterisks indicate decreases in fungal metabolism (= higher anti-fungal activity) and pound signs in brackets indicate increases in fungal metabolism (= less anti-fungal activity).

A PA14 mutant unable to produce pyoverdine or pyochelin (PA14 pvdD-pchE-; double siderophore mutant) was used to exclude pyoverdine effects from our experimental system and look for possible other *P. aeruginosa* anti-fungal molecules. The PA14 wild-type and the double siderophore mutant both showed about equal growth in RPMI and equally enhanced growth in SSPM. Filtrates of the double siderophore mutant and PA14 wild-type were prepared in RPMI or SSPM. The anti-fungal activity of RPMI filtrates was examined in RPMI (Figure 6A), and SSPM filtrates were analyzed in SSPM (Figure 6B). The double siderophore mutant filtrate showed less anti-fungal activity than the wild-type filtrate in RPMI (Figure 6A), as well as in SSPM (Figure 6B), indicating that the loss of siderophores attenuated anti-fungal activity in both media. The mutant’s filtrates, no matter the medium it was prepared in, appeared more anti-fungal when tested in SSPM.

**Figure A2:**
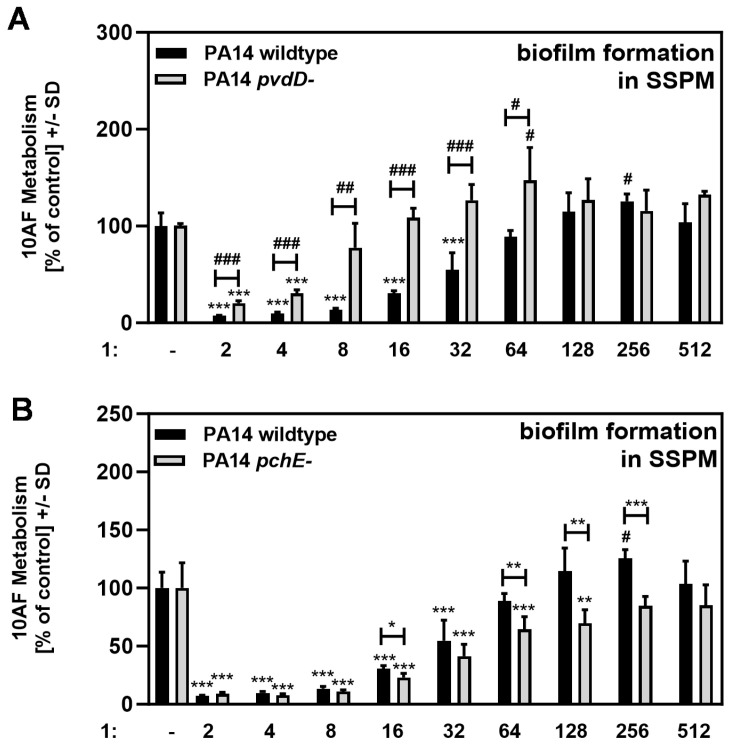
Anti-fungal activity of pyoverdine and pyochelin single mutants in RPMI and SSPM. 10AF conidia (10^5^ conidia/mL) were incubated with PA14 wild-type, pyoverdine mutant (PA14 *pvdD-*), or pyochelin mutant (PA14 *pchE-*) filtrate in SSPM in 2-fold steps (final concentrations: 1:2 to 1:256). (**A**) compares wild type to the pyoverdine mutant; (**B**) compares wild type to the pyochelin mutant. Assay plates were incubated at 37 °C for 16 h. Fungal metabolism was measured by XTT assay. Metabolism in the presence of medium alone was regarded as 100% and compared to each strain and filtrate dilution in the same medium. Comparisons without brackets were performed by 1-way ANOVA. All other comparisons were performed by unpaired *t*-test as indicated by the ends of the brackets. Statistical analysis: one, two, or three asterisks or pound signs = *p* ≤ 0.05, *p* ≤ 0.01, or *p* ≤ 0.001, respectively. Asterisks indicate decreases in fungal metabolism compared to medium control (= higher anti-fungal activity) and pound signs indicate increases in fungal metabolism, comparing RPMI vs. SSPM (= less anti-fungal activity).

We repeated the experiment in SSPM with PA14 mutants that were deficient in only pyoverdine (PA14 pvdD-; Figure A2, part A) or pyochelin (PA14 pchE-; Figure A2, part B). The pyoverdine mutant displayed great losses of activity in SSPM, whereas the pyochelin mutant approximated the wild type in SSPM with regard to IC50. This result confirmed that the loss of pyoverdine, not pyochelin, was mostly responsible for the attenuated anti-fungal activity in SSPM.

In the next step, the double siderophore mutant filtrate, prepared in RPMI or SSPM, was analyzed for anti-fungal activity after mixing with conidia in the opposite medium to allow for the same assay conditions (RPMI and SSPM, each 50% of volume). Under these conditions, the anti-fungal activity of each filtrate is solely dependent on anti-fungal molecules in the filtrates and not on differences in nutrients for the growing fungus. Under such conditions, the double siderophore mutant filtrate prepared in SSPM, but not in RPMI, showed significant anti-fungal activity (Figure 6C). In conclusion, the mutant *P. aeruginosa* produced anti-fungal molecules other than pyoverdine when the filtrate was prepared in SSPM that were not, or to a lesser degree, produced in RPMI.

### 3.6. Anti-Fungal Molecules Are Produced at a Higher Yield in SSPM

**Figure 7:**
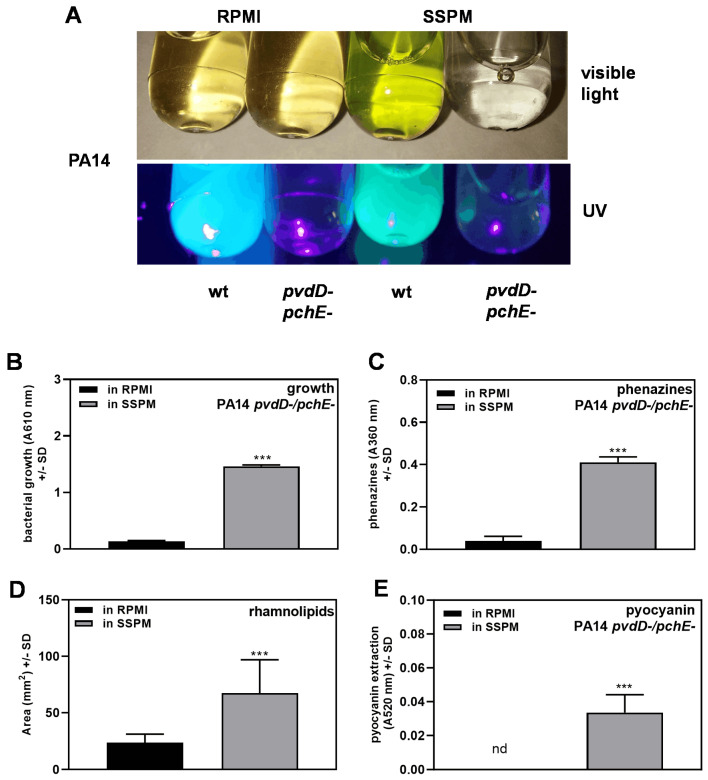
Anti-fungal molecules are produced at a higher yield in SSPM. PA14 wild type and double siderophore mutant (PA14 pvdD-pchE-) bacteria [5 × 10^7^ cells/mL] were incubated in RPMI or SSPM at 37 °C for 24 h. Filtrates were examined under visible light and UV light (**A**). Bacterial growth was measured by absorption of the cultures at 610 nm vs. their respective sterile medium as a background control (**B**). Phenazine (**C**), pyocyanin (**D**), and rhamnolipid contents (**E**) in PA14 pvdD-pchE- filtrates were determined. Comparisons: RPMI (black bars) vs. SSPM (grey bars). Statistical analysis: unpaired *t*-test; three asterisks = *p* ≤ 0.001.

When wild-type PA14 and its double siderophore mutant were used to produce filtrates in RPMI and SSPM, both strains did not produce colored filtrates in RPMI, whereas wild-type filtrate in SSPM appeared green in visible light (Figure 7A, upper part). Under UV light, the wild type in both media showed pyoverdine fluorescence, whereas the double siderophore mutant in both media did not (Figure 7A, lower part). The green color of the wild-type filtrate in SSPM remained unchanged after an attempt of chloroform extraction, suggesting that the green color was not caused by pyocyanin.

In order to determine which molecules other than pyoverdine might contribute to the increased anti-fungal activity of the double siderophore mutant in SSPM, we compared growth and phenazine, pyocyanin, and rhamnolipids production in RPMI and SSPM cultures. Our results show that the double siderophore mutant grew much better in SSPM than in RPMI (Figure 7B), as previously mentioned, and its SSPM filtrate contained more phenazines (Figure 7C), rhamnolipids (Figure 7D), and pyocyanin (Figure 7E). The amounts of pyocyanin found after extraction were very low, although significantly higher in SSPM filtrates. All of these molecules are anti-fungal and, alone or in combination, are potential contributors to increased anti-fungal activity in SSPM.

### 3.7. Rhamnolipids and Pyocyanin, but Not PQS, Have Stronger Anti-Fungal Activity in SSPM

**Figure 8:**
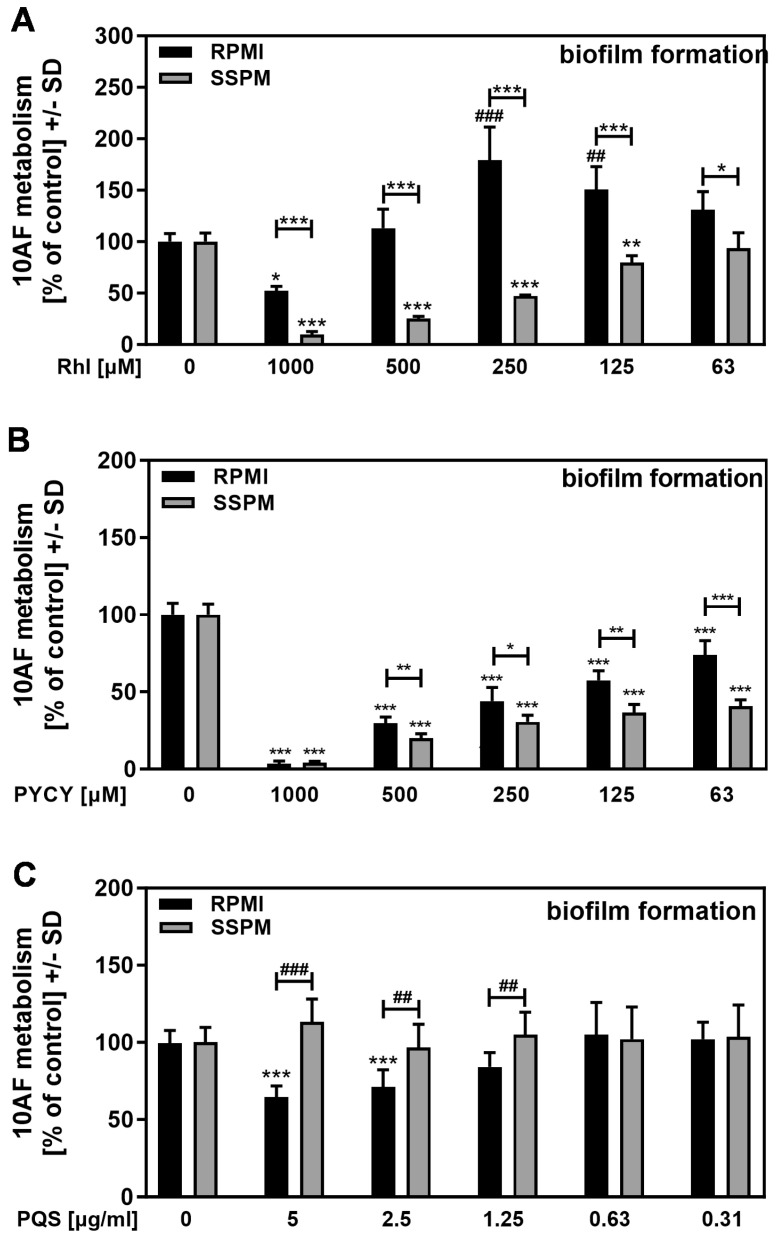
Rhamnolipids and pyocyanin, but not PQS, have stronger anti-fungal activity in SSPM. 10AF conidia (10^5^ conidia/mL) were incubated in RPMI or SSPM with or without rhamnolipids (**A**) (Rhl; final concentrations 1000–63 µM), pyocyanin (**B**) (PYCY; final concentrations 1000–63 µM), or PQS (**C**) (final concentrations 10–0.63 µg/mL). Assay plates were incubated at 37 °C for 16 h. Fungal metabolism was measured by XTT assay. Comparisons without brackets were performed by 1-way ANOVA. Metabolism in the presence of medium alone was regarded as 100% and compared to each dilution in the same medium. All other comparisons were performed by unpaired *t*-test as indicated by the ends of the brackets. Statistical analysis: one, two, or three asterisks or pound signs = *p* ≤ 0.05, *p* ≤ 0.01, or *p* ≤ 0.001, respectively. Asterisks indicate decreases in fungal metabolism (= higher anti-fungal activity) and pound signs indicate increases in fungal metabolism (= less anti-fungal activity).

When pure molecules were examined for their anti-fungal activity in RPMI vs. SSPM, rhamnolipids (Figure 8A) and pyocyanin, a member of the phenazine family (Figure 8B), showed higher anti-fungal activity in SSPM, indicating that these molecules are likely responsible for the observed higher anti-fungal activity of the double siderophore mutant in SSPM. This was also the medium where more of the molecules were produced (Figure 7D,E). PQS in SSPM did not show higher anti-fungal activity, but it even interfered with anti-fungal activity when compared to the observed results in RPMI (Figure 8C), resembling our findings for pyoverdine (Figure 5A).

## 4. Discussion

Various studies have indicated that certain regions of the CF lung, e.g., where micro-hemorrhages occur, are rich in iron, whereas others, e.g., mucus plugs, may be iron-depleted [7]. RPMI, used in clinical testing as per the CLSI, is iron-poor and therefore does not fully reflect the situation in CF lungs. In our present studies, we compared microbial interaction in RPMI to interaction in SSPM in an effort to more closely approximate the situation in the overall milieu in CF lungs.

We observed that both Pa and Af grow more (Figure 2A) and quicker (Figure 3) in SSPM than in RPMI. Both organisms utilize iron via their respective siderophores, foremost pyoverdine for Pa and SidA for Af [36,37]. In Pa filtrates produced in SSPM, more pyoverdine was detected compared to RPMI filtrates (Figure 2B), but when normalized to bacterial growth, Pa produced less pyoverdine per bacterium in SSPM (Table 2). This can be explained by the depressing effect of Fe^2+^ in SSPM on the production of iron-acquiring siderophores [10].

When the production by Pa of molecules in Pa filtrates was performed in the same medium as in the assay for inhibition of Af (both RPMI or both SSPM), the Pa filtrates in RPMI were more anti-fungal than the filtrates prepared in SSPM (Figure 4B), despite the RPMI filtrates having less pyoverdine (Figure 2B). This suggested that SSPM dampens the anti-fungal activity of Pa filtrates or protects the fungus from pyoverdine.

To unravel this further, we equalized the medium used for the inhibition assay by studying Pa filtrates prepared in RPMI against conidia suspended in SPPM, and the assay was performed in SPPM; conversely, we studied filtrates prepared in SSPM against conidia suspended in RPMI, with the assay performed in RPMI. Thus, there were now equal quantities of RPMI and SSPM in all the assays (standardized, mixed media assay (SMMa)). The SMMa allowed for fungal growth comparable to fungal growth in SSPM (Figure 4A). Under these conditions, the Pa filtrates prepared in SSPM were more anti-fungal than the filtrates prepared in RPMI (Figure 4C). However, with these assays now uniformly in 50% SSPM, anti-fungal activity was dampened, compared to the previous studies, whether the Pa filtrates were prepared in RPMI or SSPM (Figure 4B vs. Figure 4C). This suggests that SSPM decreases pyoverdine activity rather than protecting the fungus, which grew equally in SSPM vs. SMMa (Figure 4A).

We then tested pure pyoverdine and found it to indeed be less anti-fungal in SSPM than in RPMI (Figure 5A), supporting our conclusion that pyoverdine in SSPM filtrates must be dampened in its activity. Since pyoverdine was less anti-fungal in SSPM, despite the Pa filtrates prepared in SSPM being more anti-fungal in the SMMa studies (as just described), this suggested that Pa must produce anti-fungal molecules other than pyoverdine in SSPM, and/or the other anti-fungals produced are more potent inhibitors in SSPM.

We further confirmed that Fe^2+^ at concentrations found in SSPM enhances the growth of Af in RPMI (Figure 5B). However, if the increased Af growth in Fe^2+^-supplemented RPMI was normalized compared to growth in standard RPMI, the added Fe^2+^ decreased the anti-fungal activity of pyoverdine in RPMI (Figure 5C). This can explain why pyoverdine and Pa filtrates are less anti-fungal when prepared in SSPM, since SSPM contains Fe^2+^. Iron attaching to pyoverdine would decrease pyoverdine’s ability to inhibit a fungal competitor by denying iron to the fungus.

Filtrates of a Pa mutant lacking the ability to generate the two principal Pa siderophores, pyoverdine and pyochelin (both of which are anti-fungal [8])*,* were studied to gain insight into the mechanisms at play. The mutant was less anti-fungal than filtrates of wild-type Pa when the filtrates were prepared in RPMI (Figure 6A) or SSPM (Figure 6B) and studied in the assay in the same medium in which the filtrates were prepared. We noted that the mutant’s filtrates appeared more anti-fungal when these studies were performed in SSPM compared to RPMI (Figure 6A vs. Figure 6B). We also tested mutants lacking either pyoverdine (PA14 *pvdD-*) or pyochelin (PA14 *pchE-)* and could confirm that the lack of pyoverdine (Figure A2, part A), but not pyochelin (Figure A2, part B), was responsible for the loss in anti-fungal activity of the double mutant compared to the wild type (Figure 6B).

When these studies were performed in an “SMMa” fashion, so that the assay conditions were the same for the different filtrates as described above, the mutant’s filtrates prepared in SSPM were anti-fungal, but not when prepared in RPMI (Figure 6C). Therefore, we concluded that, lacking siderophore production, the mutant must produce anti-fungals other than pyoverdine in SSPM.

We then confirmed the absence of pyoverdine content in double (pyoverdine + pyochelin) mutant filtrates (Figure 7A) and found the mutant’s increased growth (Figure 7B) and increased production of phenazines (Figure 7C), biosurfactants (Figure 7D), and pyocyanin (Figure 7E) in SSPM compared to RPMI. This reasoning would also apply to the wild type, as suggested in the SMMa experiments with the wild type described above, where wild-type Pa filtrates were more anti-fungal when prepared in SSPM, despite the lessened potency of pyoverdine in SSPM. An anti-fungal molecule overrepresented in SSPM filtrates could be responsible for the observed increased anti-fungal activity of SSPM filtrates in the absence of pyoverdine.

We also found that pure rhamnolipids (Figure 8A) or pyocyanin (Figure 8B) were more anti-fungal when presented in SSPM than in RPMI. These Pa products could contribute to a greater anti-fungal activity of Pa in iron-rich parts of CF lungs. It also has to be kept in mind that such anti-fungals are over-produced in iron-rich SSPM and, hence, possibly in iron-rich parts of CF lungs where pyoverdine activity is dampened. It has already been shown that bacteria in SSPM also produce large amounts of PQS [20], another Pa molecule that, at certain concentrations, is anti-fungal but, as we have shown, can also deliver iron to the fungus [11]. PQS showed reduced activity in SSPM (Figure 8C), resembling the siderophore pyoverdine (Figure 5A), but to a lesser degree.

Possible limitations to our study are as follows: (a) there may be molecules important for microbial competition present in CF sputum that are not represented in SSPM; (b) as mentioned, there are variations in the molecular contents in different portions of CF lung, so the ratio of microbial molecules present could vary from site to site; (c) what is relevant to CF sputum may not be relevant to sputum in other clinical situations where these two pathogens also co-exist (e.g., lungs of cancer patients or transplant patients); (d) CF sputum content may be changing in the era of CFTR-manipulating drugs; and (e) CF sputum content may change in the wake of antimicrobial use in a patient.

## 5. Conclusions

Pa in SSPM (and presumably in the airways milieu which SSPM mimics) produces higher amounts of anti-fungal molecules other than pyoverdine, compensating for reduced pyoverdine activity; moreover, Af is also more sensitive to these other molecules in that milieu. Thus, SSPM appears to help illuminate the complexities of Pa–Af intermicrobial competition in the milieu of CF lungs.

## Figures and Tables

**Table 1:** Organisms used in this study.

Strain	Description	Ref.
10AF	*A. fumigatus* common laboratory strain	[25,26]
PA14(UCBPP-PA14)	*P. aeruginosa* common laboratory strain; parental strain of all PA14 mutants studied	[27,28]
PA14 *pvdD-pchE-*	PA14 Pyoverdine–pyochelin double siderophore mutant	[28,29]
PA14 *pvdD-*	Loss of pyoverdine (siderophore)	[30]
PA14 *pchE-*	Loss of pyochelin (siderophore)	[30]

**Table 2:** Bacterial product measurements and normalization to growth (mean values of n = 4).

Strain	OD610 Bacterial Growth	OD405 Pyoverdine	OD405/OD610 Relative Production
PA14 (RPMI)	0.259	0.795	3.08
PA14 (SSPM)	1.315	1.864	1.42

One example of several experiments is shown.

## Data Availability

Data supporting the reported results can be obtained from the corresponding author.
